# Identifying Patients without a Survival Benefit following Transfemoral and Transapical Transcatheter Aortic Valve Replacement

**DOI:** 10.3390/jcm10214911

**Published:** 2021-10-24

**Authors:** Daniela Geisler, Piotr Nikodem Rudziński, Waseem Hasan, Martin Andreas, Ena Hasimbegovic, Christopher Adlbrecht, Bernhard Winkler, Gabriel Weiss, Andreas Strouhal, Georg Delle-Karth, Martin Grabenwöger, Markus Mach

**Affiliations:** 1 Department of Cardio-Vascular Surgery, Klinik Floridsdorf and Karl Landsteiner Institute for Cardio-Vascular Research, 1210 Vienna, Austria; daniela.geisler@gesundheitsverbund.at (D.G.); bernhard.winkler@gesundheitsverbund.at (B.W.); martin.grabenwoeger@gesundheitsverbund.at (M.G.); 2 Department of Coronary and Structural Heart Diseases, The Cardinal Stefan Wyszyński Institute of Cardiology, 04-628 Warsaw, Poland; piotr.rudzinski@ikard.pl; 3 Department of Cardiac Surgery, Medical University Vienna, 1090 Vienna, Austria; martin.andreas@meduniwien.ac.at (M.A.); ena.hasimbegovic@meduniwien.ac.at (E.H.); 4 Imperial College London, London SW7 2AZ, UK; waseem.hasan15@imperial.ac.uk; 5 Department of Internal Medicine II, Division of Cardiology, Vienna General Hospital, 1090 Vienna, Austria; 6 Imed19-Privat, Private Clinical Research Center, Chimanistrasse 1, 1190 Vienna, Austria; c.adlbrecht@imed19.at; 7 Department of Vascular Surgery, Klinik Ottakring, 1160 Vienna, Austria; gabriel.weiss@gesundheitsverbund.at; 8 Medical Faculty, Sigmund Freud University, 1020 Vienna, Austria; 9 Department of Cardiology, Klinik Floridsdorf and the Karl Landsteiner Institute for Cardiovascular & Intensive Care Research Vienna, 1210 Vienna, Austria; andreas.strouhal@gesundheitsverbund.at (A.S.); georg.delle-karth@gesundheitsverbund.at (G.D.-K.)

**Keywords:** futility, TAVI, TAVR, SAVR

## Abstract

Transcatheter aortic valve replacement (TAVR) offers a novel treatment option for patients with severe symptomatic aortic valve stenosis, particularly for patients who are unsuitable candidates for surgical intervention. However, high therapeutical costs, socio-economic considerations, and numerous comorbidities make it necessary to target and allocate available resources efficiently. In the present study, we aimed to identify risk factors associated with futile treatment following transfemoral (TF) and transapical (TA) TAVR. Five hundred and thirty-two consecutive patients (82 ± 9 years, female 63%) who underwent TAVR between June 2009 and December 2016 at the Vienna Heart Center Hietzing were retrospectively analyzed to identify predictors of futility, defined as all-cause mortality at one year following the procedure for the overall patient cohort, as well as the TF and TA cohort. Out of 532 patients, 91 (17%) did not survive the first year after TAVR. A multivariate logistic model identified cerebrovascular disease, home oxygen dependency, wheelchair dependency, periinterventional myocardial infarction, and postinterventional renal replacement therapy as the factors independently associated with an increased one-year mortality. Our findings underscore the significance of a precise preinterventional evaluation, as well as illustrating the subtle differences in baseline characteristics in the TF and TA cohort and their impact on one-year mortality.

## 1. Introduction

Although transcatheter aortic valve replacement (TAVR) has made it possible to treat patients that were deemed high- or extremely high-risk in the context of conventional heart surgery, the central question that still has not been sufficiently explored is whether certain risk factors will preclude the patients from benefiting from the procedure.

In the last few years, TAVR has become a mainstay in the treatment of severe symptomatic aortic stenosis, yet optimizing the periinterventional management through adequate patient selection and preventing complications associated with poor outcome remains pivotal. The number of TAVR procedures is expected to keep rising due to the growing elderly population but also as a result of an increase in the number of TAVR interventions in the low-risk and intermediate-risk population, as well as the increased number of centers performing the procedures at higher volumes. This increase is attributable to the results of the randomized-controlled PARTNER3 and EVOLUT low-risk trials that managed to demonstrate a non-inferiority of TAVR in the low-risk patient collective, with regard to both safety and efficacy [1,2,3,4,5].

However, the ever-expanding role of TAVR in the treatment of severe symptomatic aortic stenosis, combined with the high costs to the healthcare system and the high level of expertise required, will inevitably bring the issue of cost-effectiveness to the forefront in the coming years [6,7,8]. Optimizing patient selection and preventing complications associated with a poor outcome will be crucial steps in ensuring the ideal allocation of scarce healthcare resources to patients who are most likely to benefit from their use.

While commonly used risk scores have proved their utility in identifying low-risk patients eligible for cardiac surgery, they are associated with numerous limitations in accurately predicting outcomes after TAVR, including an inability to adequately account for co-morbidities, frailty, and predicted mortality [2,9,10,11,12,13].

Thus, the objective of this study was to identify clinically relevant predictors of futility within the first year after TAVR, with an underlying aim of improving the effectiveness of preinterventional screening and enhancing the vigilance for certain risk factors to further improve survival and reduce the financial strain on the healthcare system.

## 2. Methods

### 2.1. Design and Patients

Between June 2009 and December 2016, 532 consecutive patients (female 63%) undergoing TAVR for symptomatic aortic valve stenosis at the Heart Center Hietzing in Vienna were prospectively enrolled in the Vienna Cardiothoracic Aortic Valve Registry (VICTORY). The mean age was 82 ± 9 years. As an early adopter of TAVR and national referral center for transapical (TA) procedures, the TAVR procedure was performed equally via the transfemoral (TF; *n* = 266) or the TA access route (*n* = 266). Operative mortality risk was calculated using the logistic European System for Cardiac Operative Risk Evaluation (EuroSCORE) and the EuroSCORE II [14,15]. The eligibility for TAVR was assessed by a multi-disciplinary heart team consisting of cardiothoracic surgeons, cardiologists, anesthesiologists, and radiologists. The institutional diagnostic protocol for patients with aortic valve stenosis follows the general recommendations stated in the current ESC/EACTS guidelines for the management of valvular heart disease [2].

The study was approved by the Ethics Committee of Vienna (EK18-027-VK). All recruited patients signed an informed consent prior to the enrollment in the registry. Subsequently, a retrospective analysis of the patient characteristics including medical history, length of hospital stay, echocardiographic information, clinical and interventional data, and mortality was carried out in order to identify independent predictors of 1-year mortality. Mortality data, including the cause of death, was obtained by examining hospital records and via an inquiry to the Federal Institute for Statistics Austria.

### 2.2. Procedure

The preinterventional assessment included preinterventional echocardiography as well as multislice computed tomography examinations for all patients. The interventions were performed in a standard fashion by the institution’s heart team and have been described in detail before [16]. Balloon pre- and post-dilatation was performed at the operator’s discretion. Different generations of transcatheter heart valves (THV) by Edwards Lifesciences (Edwards Lifesciences, Irvine, CA, USA), Medtronic (Medtronic, Minneapolis, MN, USA), JenaValve (JenaValve Technology GmbH, Munich, Germany), and Symetis (Symetis SA, a Boston Scientific company, Ecublens, Switzerland) were used for TAVR procedures. The choice of valve size was based on a multislice computed tomography scan and echocardiography performed prior to the intervention. General anesthesia was used for all TA-TAVR procedures and for TF procedures performed before September 2014. Following a change in the institutional standard operating procedures, TF-TAVR was performed under conscious sedation after this time, whenever applicable.

### 2.3. Endpoints

The primary endpoint of this analysis was futility, defined as all-cause mortality at one year following TAVR, regardless of the patient’s subjective quality of life indicators or functional parameter improvement. The secondary endpoints, as determined by the Valve Academic Research Consortium (VARC)-2 document, were compared between survivors and non-survivors at one year following TAVR [17]. Cerebrovascular disease (CVD) was diagnosed using preinterventional doppler, and cerebrovascular accident was diagnosed according to VARC-2 criteria.

### 2.4. Statistical Analysis

The study population was separated into two cohorts: patients for whom treatment with TAVR was futile, i.e., who did not survive the first year, and patients who lived past the one-year post-TAVR timepoint. Further stratification has been performed according to the chosen access strategy. Dichotomous parameters were expressed as absolute and relative frequencies and continuous variables as median and median deviation of the median (MAD). A univariate Cox regression analysis was used to identify preinterventional, peri-interventional, and postinterventional factors, which were associated with a change in the hazard ratio. Significant preinterventional factors were finally included in a multivariate Cox regression analysis to identify those with a true impact on futile TAVR treatment.

Statistical analysis was completed using RStudio (Version 1.4.1717, 2009–2021 RStudio PBC), the reported *p*-values are 2-sided with an alpha level set at <0.05 for statistical significance.

## 3. Results

### 3.1. Baseline Characteristics

A detailed comparison of the baseline characteristics is presented in Table 1.

### 3.2. Preinterventional Parameters of Survival in the First Year

In a univariate Cox regression analyses, the TA approach, EuroSCORE II, chronic obstructive pulmonary disease (COPD), peripheral vascular disease (PVD), CVD, home oxygen dependence, and the mean and maximum pressure gradient across the aortic valve demonstrated to be of significant influence on the primary study endpoint in the overall patient cohort, respectively (Table 2). In the TF subgroup, only wheelchair dependence was a significant negative factor of survival in the first year (Table 2). In the TA subgroup, the logistic EuroSCORE, the EuroSCORE II, peripheral vascular disease, CVD, and home oxygen dependence showed significantly increased hazard ratios (Table 2).

CVD remained associated with futile treatment following TAVR in the multivariate Cox regression analyses (Supplementary Table S1) in the combined access cohort as well as the TA subgroup. Home oxygen dependence remained statistically significant in the TA subgroup.

### 3.3. Interventional Factors of Survival in the First Year

Conversion to open surgery, total hours in the intensive care unit (ICU), total hours ventilated, and length of stay after TAVR showed to be significant in the combined access cohort in the univariate Cox regression analysis (Table 3).

In the TF subgroup, applied contrast medium dosage and total ICU hours were significantly associated with futile TAVR treatment. In the TA subgroup, predillatation, conversion to open surgery, total ICU hours, total hours ventilated, and length of in-hospital stay after TAVR showed significantly increased hazard ratios (Table 3).

After multivariate Cox regression analysis total hours in the ICU and length of stay after TAVR remained independently associated with futile treatment in the TF and the TA groups, respectively (Supplementary Table S2)

### 3.4. Adverse Events

In the combined access cohort, the VARC-2 composite endpoints of device success and the 30-day combined safety endpoint as well as acute kidney injury, new atrial fibrillation, reoperation for non-cardiac problems, reoperation for bleeding/tamponade, major bleeding complications, new renal replacement therapy, neurological adverse events, and peri- or postinterventional myocardial infarction were associated with futile treatment within the first year after TAVR following univariate Cox regression analysis (Table 4).

Major bleeding complications, neurological adverse events, and the maximum pressure gradient across the TAVR prosthesis were significantly associated with futile TAVR treatment in the TF subgroup in the univariate regression model (Table 4).

Device success, the 30-day combined safety endpoint, acute kidney injury, new atrial fibrillation, reoperation for non-cardiac problems, reoperation for bleeding/tamponade, pneumonia under antibiotic treatment, major bleeding complications, new renal replacement therapy, major vascular complication, neurological adverse events, reoperation for valvular dysfunction, and myocardial infarction showed to be negative factors of survival within the first year after TA-TAVR (Table 4).

Multivariate Cox regression analysis identified an increased risk of futility with new renal replacement therapy in the combined access cohort. Major bleeding complication and myocardial infarction were independently associated with futility following TA-TAVR (Supplementary Table S3).

## 4. Discussion

The reported 1-year mortality rates following TAVR range between 1% and 14.5%, depending on whether the patients belong to the low or intermediate risk group [3,5,18,19]. This suggests that, although this treatment option is not as invasive as surgical aortic valve replacement (SAVR) and carries many associated benefits, a considerable number of patients will fail to show signs of clinical improvement and are at an increased risk of dying shortly after the procedure. Depending on the patient’s comorbidities and further clinical factors, the choice of access is most often made between the TF and the TA access site. The latter remains the main alternative access route in most hospitals worldwide despite other potentially less invasive access route strategies. The respective patient cohorts differ both in their preclinical makeup and in the range of postinterventional adverse events and outcomes. Thus, in our study, we attempted to highlight some of the most important risk factors for futility for the combined access patient collective, on the one hand, but more importantly, we considered these factors for both access sites independently in order help optimize patient selection, access site allocation, promote a fast-track post-operative course, early discharge, and thus, improve overall survival. Thus, in our work, we were not only able to validate certain parameters that have been demonstrated to be significant predictors for futility in existing research, but based on our extensive database structure, we were also able to identify several new parameters that have received little attention in the past and have not yet found their way into clinical trials.

### 4.1. Clinical Baseline Characteristics

Our study has been able to confirm that TA access is an independent predictor of 1-year mortality following TAVR, which has been demonstrated by Mohr et al. [20]. Whilst in the TF-TAVR group, the 1-year mortality was 12.0%; in the TA-TAVR group, the 1-year mortality showed to be 22.2%. Expectedly, the logistic EuroSCORE and the EuroSCORE II correlated well with the risk of futile treatment following TA-TAVR in the first year, a mean EuroSCORE II of 4.6 means a hazard ratio (HR) of 1.4 (=1.069^4.6^), a EuroSCORE of 10 a HR of 1.9 (=1.069^10^), and a EuroSCORE II of 15 a HR of 2.7 (=1.069^15^). Therefore, they need to be interpreted with due caution especially in combination with the identified risk factors of futility during the preinterventional assessment.

The major indications for a TA approach are primarily the inability to perform the valve replacement through a TF approach due to small vessel size or their prominent tortuosity or calcification, a history of previous vascular interventions in the aorta, the iliac or femoral arteries, or a pronounced obesity with deep vessels and, thus, a high risk of vascular complications [21,22,23,24,25,26]. In contrast, the list of contraindications for a TA access procedure is rather short and mostly revolves around a reduced ejection fraction or thrombotic material in the apex area [21,22,23].

We identified PVD to occur in one fifth of the patients in the combined access cohort with a HR of 1.7 for futile treatment. Interestingly, patients with pronounced PVD were also found to be less likely to benefit from the TA access. Since atherosclerosis is known to be a systemic disease, it is mostly not limited to a single artery territory, but spread to the whole organism. CVD, on the other hand, puts the patient at a 2.3-fold higher risk of undergoing futile TA-TAVR treatment and affects every sixth patient. After multivariate Cox regression analysis, CVD remained the strongest predictor for 1-year mortality following TAVR in the combined access cohort and the TA access group. Thus, we advocate that patients with a combination of CVD and pronounced PVD be subjected to a more stringent risk–benefit analysis prior to undergoing TA-TAVR.

One of the most prominent novel findings in our TF-TAVR cohort is wheelchair use as a predictor of TF-TAVR futility that is currently not regularly considered and evaluated when planning TAVR interventions and choosing the access site. It should also be questioned whether and to what extent this patient collective is likely to subjectively benefit from an increase in their physical resilience. Thus, this finding warrants further studies in this particular patient collective.

Although it is well established in the recent literature that COPD as a concomitant risk factor does not necessarily lead to a worse outcome after TA-TAVR, our study demonstrated that pronounced pulmonary oxygenation impairment resulting in home oxygen dependence is significantly associated with futile treatment after TA-TAVR [27]. However, it has to be pointed out that home oxygen dependence is a very rare clinical condition with an overall incidence of less than 2%, yet should be incorporated in the preinterventional decision making process when present.

Additionally, a lower preinterventional mean and maximum pressure gradient across the aortic valve such as is often encountered in patients with low-flow–low-gradient aortic stenosis were associated with futile TAVR treatment in the overall TAVR group. This finding is consistent with evidence from the recent literature [PMID: 31000012, PMID: 33289422] and emphasizes the importance of correctly interpreting long-term myocardial sequelae rather than assessing LVEF alone in preinterventional risk assessment. As the overall incidence paradoxical low-flow–low-gradient aortic stenosis was rather low (<1% in the entire cohort), our finding supports the hypothesis that patients with a low LVEF and consecutively low pressure gradients across the aortic valve display worse postinterventional outcome after TAVR than patients with a low LVEF that can nevertheless generate high gradients across the aortic valve [28,29,30]. The absence of the binary variable low-flow–low-gradient aortic stenosis in our cohort as a significant predictor of futile treatment at 1 year indicates that pressure gradients are likely to have a higher sensitivity due to the relatively high cut-off value of 50% for LVEF in the current guidelines. Substratification within this patient population based on their LVEF could potentially provide new, important conclusions in future analyses.

### 4.2. Interventional Factors

Although technical procedure-related problems are diverse and hard to predict, our results once again underpin the importance of avoiding a conversion to open heart surgery, most importantly through a precise preinterventional risk assessment. The severity of this rare complication is perhaps best illustrated by the fact that only three of the nine patients who underwent a conversion to SAVR survived past the 1-year timepoint. The procedures documented in this study were all undertaken in either a cardiac catheterization laboratory or a standard operating room (OR). The implementation of TAVR in a hybrid OR may provide distinct advantages such as prompt treatment of unplanned procedures or procedures requiring circulatory support, as well as the optimal infrastructural background for the collaboration between cardiologists and cardiothoracic surgeons. At our institution, all TAVR procedures are now performed in the hybrid OR. This approach seems to be the optimal way to maximize the safety and comfort of the patient and enable the staff to perform the most complex bail-out procedures at maximum speed and efficiency. In line with already available data, some of the more prominent drivers were increased ventilation times, prolonged ICU and hospital stay in the combined access cohort [12], and total hours at the ICU in the TF-TAVR group, respectively. However, the factor time must not be regarded as a cause rather than effect and relates to severely ill patients. Consequently, our data further indicate that a prolonged procedure time was associated with a worse outcome in the TA access cohort, with this increase generally attributable to either increased difficulty of the surgical procedure itself (due to patient-specific anatomic conditions) or intraoperative complications.

With respect to the choice of anesthesia, opting for conscious sedation rather than general anesthesia has gained popularity over the last couple of years, especially in patients with ventilatory disorders and difficult airways. Patients with chronic lung disease are particularly prone to prolonged ventilation times, which negatively impact the weaning process and extend the length of their ICU and hospital stay [31]. Thus, ventilation times must be interpreted as a surrogate parameter for several clinical factors including preinterventional morbidity, interventional complexity, and postinterventional complications, as well as frailty. Another important contributing factor towards a prolonged postinterventional course is the increased risk of pneumonia requiring antibiotic treatment in the transapical cohort. Furthermore, home oxygen dependence as a predictor of futility in the TA-TAVR group is likely to result in longer ventilation times and a correspondingly prolonged ICU stay. It is important to point out that the TF patients whose data were collected for this study were not routinely treated under conscious sedation, a standard in our institution since 2014.

### 4.3. Success Factors and Adverse Events

As expected, the VARC-2 composite endpoints of device success and safety at thirty days are closely related to the risk of futility after TAVR. Other adverse events that were identified as significant predictors of TAVR futility include acute kidney injury, postinterventional renal replacement therapy, major bleeding complications, new-onset atrial fibrillation reoperation for non-cardiac problems or reoperation for bleeding or cardiac tamponade, neurological adverse events, and myocardial infarction [32]. Acute kidney injury is one of the most recognized complications following TAVR and plays a key role in short- and long-term mortality. New renal replacement therapy was associated with a six-fold increased risk of futility in the combined access cohort and a nearly 10-fold increase in risk in the TA-TAVR group. This finding particularly stresses the importance of future research being directed towards preventive strategies to reduce the incidence of acute kidney injury following TAVR. While major bleeding complications occurred in both the transfemoral and transapical cohorts, they resulted in an associated hazard ratio of 4.2 in the combined access cohort, highlighting the importance of careful postinterventional hemostasis. Neurological adverse events following TAVR displayed as one of the overarching risk factors for futile procedures in both cohorts, and thus, we have to emphasize the importance of developing and expanding periinterventional neuroprotection protocols and improving the preinterventional risk assessment in patients with a history of cerebrovascular disease with regard to stroke prevention. These findings are in line with results presented in the recent literature [24,33]. However, in the multivariate analysis, major bleeding complications and neurological adverse events were outperformed by the 30-day composite safety endpoint.

Although periinterventional myocardial infarction occurred with an overall incidence of only 0.6%, it should be noted that this pivotal clinical event was associated with a 40-fold increased risk of futile treatment in the TA-TAVR cohort and, hence, warrants special consideration in high-risk settings such as valve-in-valve procedures in failed bioprostheses with outside-mounted leaflets.

As the volume of data available on the topic of TF-TAVR is high due to the increasing number of conducted interventions, it is important to point out that a standardized tool to identify patients for whom a futile intervention seems likely is still not available, and many futility predictors might not yet have been identified. In this respect, and with the ever-increasing number of TAVR patients and indications, it is important, both in order to optimize resource distribution in the healthcare system and to avoid unnecessary interventions, to work towards identifying comorbidities that might make a futile TAVR highly likely. The proportion of patients treated through a TA access site in our cohort is relatively high because of the high referral rate of patients ineligible for a TF approach, and the TF-TAVR route remains the first-choice treatment for most patients, due to its low invasiveness and good outcomes. However, it is important to recognize that the results of side-by-side comparisons of TF- and TA-TAVR are often skewed by the makeup of the patient collective [34]. To summarize, the choice of access site is more than a purely technical consideration, and it is paramount that all patients undergo a detailed preinterventional evaluation in order to choose the optimal access point and plan the intervention depending on the patient’s anatomy [21,22,23,35]. Further research should be directed towards exploring the possibility that the poor TA-TAVR-associated outcome might be improved by identifying patient subgroups that might have a high futility risk and might benefit from an entirely different access point.

## 5. Study Limitations

Futility has not yet been defined in any of the current valvular heart disease guidelines. Furthermore, there is no common agreement on which the quality of life (QOL) assessment tool should be used before and after TAVR and how “improvement” is defined. Due to the wide range of symptoms and the varying clinical state of the patients, different QOL indicators might only be applicable to or disproportionately subjectively valued by certain patient subgroups. Inherent limitations of this study are the retrospective single-center design and the fact that the assessment was based solely on clinical endpoints and available registry data. This is mostly a direct consequence of the fact that the patient collective stems from multiple regions of Austria, and further follow-up examinations mostly take place in the referring institution. The number of events in each group is small, which should be considered when establishing statistical comparisons. Technical advances, the implementation of new generation THV devices, and an inherent learning curve could have biased outcomes. Furthermore, there is currently an ever-increasing shift towards performing TAVR in a hybrid OR, whilst the interventions described in the study took place in a cardiac catheterization laboratory or a standard OR.

## 6. Conclusions

In our study, we attempted to highlight some of the most important risk factors for futility for TAVR patients on the one hand, but more importantly we considered these factors for both access sites, TF and TA, independently in order help optimize patient selection, access site allocation, promote a fast-track post-operative course, early discharge, and thus, improve overall survival. Factors were addressed in three groups according to their timely order (pre-, intra-, and post-procedure).

Our findings suggest reevaluating and expanding neuroprotection protocols for all patients following TAVR, but particularly for patients with a history of cerebrovascular disease. Furthermore, strategies to prevent major bleeding complications are of particular importance, especially in the patients treated via transapical access. With an almost two-fold increase in risk for futility after TAVR, the transapical access should be strictly restricted to patients with no viable option for percutaneous transfemoral treatment. A more detailed risk assessment of oxygen and wheelchair-dependent patients seems warranted in patients treated via transapical and transfemoral access pathways, respectively.

## Figures and Tables

**Table 1:** Baseline clinical characteristics of futile and non-futile TAVR procedures.

	Combined Access		TF-TAVR				TA-TAVR			
			Futile		Non-Futile		Futile		Non-Futile	
Demographics										
	*n* = 532		*n* = 32		*n* = 234		*n* = 59		*n* = 207	
Age, median (MAD)	82	(5.9)	84.5	(5.2)	83	(5.9)	83	(7.4)	80	(7.4)
Female, *n* (%)	335	(63)	19	(59.4)	153	(65.4)	33	(55.9)	130	(62.8)
Body mass index kg/m2, median (MAD)	25.8	(4.7)	26.2	(5.4)	25.9	(4.4)	24.8	(4.9)	26.1	(4.9)
Risk profile										
Logistic EuroSCORE, median (MAD)	15.1	(9.2)	13.6	(6.5)	14.5	(8.4)	19.3	(11.8)	15.5	(10.4)
EuroSCORE II, median (MAD)	4.6	(3.2)	4.8	(2.3)	4.3	(2.9)	6.6	(3.7)	4.6	(3.5)
Incremental risk score, median (MAD)	6	(8.9)	9.5	(9.6)	6.2	(9.1)	7	(10.4)	5	(7.4)
Chronic health conditions and risk factors ordered by its frequency										
Hypertension, *n* (%)	467	(87.8)	30	(93.8)	205	(87.6)	53	(89.8)	179	(86.5)
Dyslipidaemia, *n* (%)	320	(60.2)	19	(59.4)	123	(52.6)	43	(72.9)	135	(65.2)
Renal impairment eGFR < 60 mL/min/1.73 m^2^, *n* (%)	296	(55.6)	16	(50)	129	(55.1)	34	(57.6)	117	(56.5)
Coronary artery disease, *n* (%)	267	(50.2)	14	(43.8)	115	(49.1)	33	(55.9)	105	(50.7)
Prior PCI, *n* (%)	165	(31)	10	(31.2)	70	(29.9)	20	(33.9)	65	(31.4)
Atrial fibrillation, *n* (%)	163	(30.6)	11	(34.4)	67	(28.6)	17	(28.8)	68	(32.9)
Peripheral vascular disease, *n* (%)	106	(19.9)	3	(9.4)	24	(10.3)	24	(40.7)	55	(26.6)
Diabetes mellitus (IDDM), *n* (%)	91	(17.1)	5	(15.6)	38	(16.2)	13	(22)	35	(16.9)
Prior myocardial infarction, *n* (%)	89	(16.7)	5	(15.6)	29	(12.4)	13	(22)	42	(20.3)
Permanent pacemaker, *n* (%)	85	(16)	7	(21.9)	48	(20.5)	10	(16.9)	20	(9.7)
Previous CABG, *n* (%)	84	(15.8)	5	(15.6)	30	(12.8)	11	(18.6)	38	(18.4)
Cerebrovascular disease, *n* (%)	83	(15.6)	4	(12.5)	27	(11.5)	20	(33.9)	32	(15.5)
Cerebrovascular accident, *n* (%)	70	(13.2)	4	(12.5)	34	(14.5)	9	(15.3)	23	(11.1)
COPD, *n* (%)	66	(12.4)	4	(12.5)	15	(6.4)	14	(23.7)	33	(15.9)
Previous valve surgery, *n* (%)	50	(9.4)	1	(3.1)	26	(11.1)	5	(8.5)	18	(8.7)
Liver cirrhosis, *n* (%)	28	(5.3)	3	(9.4)	7	(3)	4	(6.8)	14	(6.8)
Home oxygen dependence, *n* (%)	8	(1.5)	1	(3.1)	4	(1.7)	3	(5.1)	0	(0)
Wheel chair dependency, *n*(%)	5	(0.9)	2	(6.2)	3	(1.3)	0	(0)	0	(0)
Renal replacement therapy, *n* (%)	4	(0.8)	0	(0)	0	(0)	1	(1.7)	3	(1.4)
Creatinine mg/dL, median (MAD)	1.1	(0.4)	1.2	(0.5)	1.1	(0.4)	1.2	(0.4)	1.1	(0.3)
Preinterventional echocardiographic data										
Low-flow–low-gradient stenosis, *n* (%)	77	(14.5)	6	(18.8)	29	(12.4)	12	(20.3)	30	(14.5)
Aortic valve area, median (MAD)	0.7	(0.1)	0.7	(0.1)	0.7	(0.1)	0.7	(0.3)	0.7	(0.1)
Mean pressure gradient, median (MAD)	45	(14.8)	43	(11.9)	45.5	(13.3)	43.5	(15.6)	45	(17.8)
Max. pressure gradient, median (MAD)	69	(20.8)	67	(16.3)	71	(19.3)	68.2	(25.6)	69	(22.2)
Peak velocity m/sec, median (MAD)	4.1	(0.6)	4	(0.5)	4.1	(0.6)	4	(0.6)	4	(0.7)
LVEF %, median (MAD)	55	(7.4)	60	(0)	60	(0)	55	(7.4)	55	(7.4)

CABG—coronary artery bypass graft; COPD—chronic obstructive pulmonary disease; eGFR—estimated glomerular filtration rate; EuroSCORE—European System for Cardiac Operative Risk Evaluation; IDDM—insulin-dependent diabetes mellitus; MAD—median deviation of the median; LVEF—left ventricular ejection fraction; PCI—percutaneous coronary intervention; TA—transapical; TAVR—transcatheter aortic valve replacement; TF—transfemoral.

**Table 2:** Univariate Cox regression analyses of futile events based on patients’ baseline characteristics of preinterventional factors. A hazard ratio (HR) above 1 increases the risk, below 1 decreases the risk of futility.

	Combined Access				TF-TAVR				TA-TAVR			
	HR	95% CI		*p*-Value	HR	95% CI		*p*-Value	HR	95% CI		*p*-Value
Demographics												
Transapical access	1.9	1.2	2.9	0.003								
Age	1.014	0.985	1.045	0.345	1.019	0.961	1.08	0.535	1.027	0.991	1.064	0.145
Male gender	1.29	0.852	1.954	0.23	1.26	0.622	2.552	0.52	1.266	0.757	2.116	0.369
Body mass index	0.988	0.948	1.03	0.56	1.019	0.955	1.088	0.565	0.967	0.916	1.021	0.227
Risk profile												
Logistic EuroSCORE	1.014	0.999	1.029	0.075	0.985	0.953	1.019	0.384	1.024	1.007	1.041	0.006
EuroSCORE II	1.039	1.002	1.078	0.039	0.972	0.894	1.058	0.514	1.069	1.024	1.116	0.003
Incremental risk score	1.008	0.986	1.03	0.5	1.003	0.966	1.042	0.876	1.011	0.985	1.038	0.414
Chronic health conditions and risk factors ordered by its frequency as in Table 1												
Hypertension	1.468	0.711	3.034	0.299	1.991	0.476	8.331	0.346	1.324	0.569	3.08	0.515
Dyslipidaemia	1.455	0.936	2.262	0.095	1.312	0.648	2.656	0.451	1.348	0.759	2.393	0.308
Renal impairment	0.962	0.636	1.453	0.853	0.82	0.41	1.639	0.574	1.033	0.616	1.731	0.903
Coronary artery disease	1.06	0.703	1.6	0.779	0.793	0.395	1.595	0.516	1.229	0.735	2.054	0.433
Prior PCI	1.091	0.705	1.689	0.696	1.023	0.484	2.16	0.953	1.122	0.655	1.924	0.675
Atrial fibrillation	1.008	0.646	1.573	0.973	1.259	0.607	2.612	0.535	0.861	0.49	1.512	0.602
Peripheral vascular disease	1.765	1.126	2.768	0.013	0.906	0.276	2.975	0.871	1.719	1.022	2.89	0.041
Diabetes mellitus	1.223	0.730	2.049	0.444	0.979	0.377	2.542	0.965	1.315	0.711	2.435	0.383
Prior myocardial infarction	1.245	0.743	2.085	0.406	1.236	0.476	3.208	0.664	1.114	0.602	2.061	0.732
Permanent pacemaker	1.215	0.717	2.058	0.47	1.101	0.476	2.545	0.822	1.591	0.806	3.142	0.181
Previous CABG	1.141	0.665	1.958	0.632	1.229	0.473	3.191	0.672	1.01	0.525	1.945	0.976
Cerebrovascular disease	2.042	1.281	3.256	0.003	1.076	0.377	3.066	0.892	2.322	1.354	3.982	0.002
Cerebrovascular accident	1.132	0.629	2.035	0.68	0.855	0.3	2.439	0.77	1.417	0.697	2.882	0.336
COPD	1.744	1.041	2.921	0.035	1.862	0.653	5.31	0.245	1.432	0.786	2.609	0.241
Previous valve surgery	0.63	0.275	1.441	0.274	0.27	0.037	1.977	0.197	0.889	0.356	2.222	0.801
Liver cirrhosis	1.545	0.715	3.34	0.269	2.564	0.781	8.417	0.121	1.057	0.383	2.917	0.915
Home oxygen dependence	3.294	1.208	8.983	0.020	1.695	0.231	12.415	0.604	6.334	1.963	20.438	0.002
Wheel chair dependency	3.402	0.838	13.818	0.087	4.976	1.188	20.844	0.028				
Renal replacement therapy	1.614	0.225	11.584	0.634					1.235	0.171	8.914	0.835
Creatinine mg/dL	1.221	0.959	1.555	0.105	1.184	0.634	2.212	0.596	1.186	0.917	1.534	0.193
Preinterventional echocardiographic data												
Aortic valve area	2.798	0.812	9.64	0.103	3.07	0.408	23.085	0.276	2.219	0.454	10.839	0.325
Mean pressure gradient	0.986	0.974	0.998	0.020	0.983	0.959	1.008	0.175	0.989	0.977	1.003	0.113
Max. pressure gradient	0.988	0.979	0.997	0.009	0.987	0.97	1.005	0.156	0.99	0.98	1	0.051
Peak velocity m/sec	0.841	0.659	1.072	0.161	0.871	0.576	1.316	0.511	0.841	0.621	1.139	0.262
LVEF %	0.988	0.971	1.006	0.186	1.003	0.974	1.034	0.826	0.981	0.959	1.004	0.107
Low-flow–low-gradient stenosis	1.509	0.901	2.528	0.118	1.581	0.651	3.842	0.312	1.397	0.741	2.633	0.302

CABG—coronary artery bypass graft; COPD—chronic obstructive pulmonary disease; EuroSCORE—European System for Cardiac Operative Risk Evaluation; LVEF—left ventricular ejection fraction; PCI—percutaneous coronary intervention; TA—transapical; TAVR—transcatheter aortic valve replacement; TF—transfemoral.

**Table 3:** Univariate Cox regression analyses of futile events based on interventional parameters. A hazard ratio (HR) above 1 increases the risk, below 1 decreases the risk of futility.

	Combined Access				TF-TAVR				TA-TAVR			
	HR	95% CI		*p*-Value	HR	95% CI		*p*-Value	HR	95% CI		*p*-Value
Dichotomic parameters												
Predillatation necessary	1.469	0.909	2.374	0.116	1.448	0.508	4.127	0.489	2.046	1.175	3.561	0.011
Balloon expanding valve	1.091	0.723	1.647	0.677	1.085	0.502	2.346	0.835	0.629	0.367	1.079	0.092
Postdillatation necessary	1.322	0.805	2.171	0.271	0.872	0.336	2.265	0.779	1.61	0.896	2.893	0.111
Conversion to open surgery	7.023	3.066	16.083	<0.001	0	0	Inf	0.998	7.166	3.075	16.697	<0.001
Unplanned V-i-V implantation	0	0	Inf	0.995	0	0	Inf	0.996	0	0	Inf	0.995
Interval scaled parameters												
Prosthesis size	0.955	0.872	1.046	0.319	0.981	0.839	1.148	0.811	1.045	0.911	1.199	0.53
Absorbed radiation	1	1	1	0.196	1	1	1	0.844	1	1	1	0.888
Contrast medium dosage	1	0.999	1.001	0.521	1.002	1.001	1.003	<0.001	1	0.998	1.002	0.776
Procedure time	1.003	1	1.006	0.072	0.999	0.989	1.01	0.885	1.003	1	1.006	0.037
Max. creatinine in 72 h	1.126	0.874	1.451	0.359	1.026	0.587	1.793	0.927	1.124	0.839	1.506	0.434
Total hours in the ICU	1.004	1.002	1.005	<0.001	1.006	1.002	1.009	0.001	1.003	1.002	1.004	<0.001
Total hours ventilated	1.007	1.005	1.009	<0.001	1.001	0.988	1.013	0.936	1.008	1.006	1.011	<0.001
Length of hospital stay	1.028	1.015	1.042	<0.001	1.019	0.993	1.046	0.151	1.038	1.019	1.058	<0.001

ICU—intensive care unit; MAD—median deviation of the median; TA—transapical; TAVR—transcatheter aortic valve replacement; TF—transfemoral, V-i-V—valve in valve.

**Table 4:** Univariate Cox regression analyses of futile events based on postinterventional adverse events. A hazard ratio (HR) above 1 increases the risk, below 1 decreases the risk of futility.

	Combined access				TF-TAVR				TA-TAVR			
	HR	95% CI		*p*-Value	HR	95% CI		*p*-Value	HR	95% CI		*p*-Value
Dichotomic parameters												
Device success	0.528	0.294	0.95	0.033	0.65	0.267	1.579	0.341	0.219	0.099	0.482	<0.001
30-day combined safety endpoint	0.117	0.078	0.178	<0.001	0.095	0.047	0.19	<0.001	0.135	0.081	0.226	<0.001
Acute kidney injury	1.764	1.083	2.873	0.023	1.413	0.611	3.267	0.419	2.196	1.205	4.003	0.01
New pacemaker implanted	0.619	0.299	1.28	0.196	0.833	0.32	2.17	0.709	0.553	0.173	1.77	0.318
New AV-block III	0.526	0.23	1.205	0.129	0.448	0.107	1.877	0.272	0.603	0.218	1.667	0.33
New atrial fibrillation	1.927	1.122	3.309	0.017	1.389	0.422	4.569	0.589	1.905	1.029	3.528	0.04
Major bleeding complication	4.267	2.657	6.852	<0.001	6.289	2.82	14.024	<0.001	3.133	1.742	5.634	<0.001
Reoperation for bleeding/tamponade	2.788	1.548	5.021	0.001	2.268	0.689	7.46	0.178	2.772	1.402	5.479	0.003
Reoperation for other cardiac problems	1.659	0.903	3.047	0.103	1.893	0.776	4.614	0.161	1.822	0.783	4.24	0.164
Reoperation for non-cardiac problems	2.453	1.387	4.339	0.002	1.809	0.432	7.584	0.417	2.267	1.202	4.276	0.011
Pneumonia under antibiotic treatment	1.843	0.926	3.669	0.082	0.374	0.051	2.739	0.333	5.042	2.378	10.688	<0.001
New renal replacement therapy	6.319	3.352	11.91	<0.001	2.914	0.696	12.201	0.143	9.582	4.636	19.808	<0.001
Major vascular complication	1.952	0.716	5.319	0.191	0	0	Inf	0.997	5.513	1.996	15.225	0.001
Neurological adverse event	3.576	1.45	8.818	0.006	4.086	1.243	13.432	0.02	4.486	1.091	18.455	0.038
Reoperation for valvular dysfunction	3.491	0.859	14.194	0.081	0	0	Inf	0.998	4.237	1.03	17.434	0.045
Myocardial infarction	8.152	2.003	33.17	0.003	0	0	Inf	0.998	41.535	9.121	189.135	<0.001
Interval scaled parameters post-implant												
Mean gradient aortic valve	0.955	0.872	1.046	0.319	0.981	0.839	1.148	0.811	1.045	0.911	1.199	0.53
Max. gradient aortic valve	1	1	1	0.196	1	1	1	0.844	1	1	1	0.888
Max. flow velocity aortic valve	1	0.999	1.001	0.521	1.002	1.001	1.003	<0.001	1	0.998	1.002	0.776

AV—atrioventricular; MAD—median deviation of the median; TA—transapical; TAVR—transcatheter aortic valve replacement; TF—transfemoral.

## Data Availability

The data presented in this study are available on reasonable request from the corresponding author.

## Supplementary Materials

The following are available online at https://www.mdpi.com/article/10.3390/jcm10214911/s1, Supplementary Table S1: Multivariate Cox regression analyses of futile events based on patients’ baseline characteristics of preoperative factors. Supplementary Table S2: Multivariate Cox regression analyses of futile events based on patients’ interventional characteristics. Supplementary Table S3: Multivariate Cox regression analyses of futile events based on patients’ VARC-2 adverse events.
