# Supplementary material for: Identifying Patients without a Survival Benefit following Transfemoral and Transapical Transcatheter Aortic Valve Replacement
Source: J Clin Med. 2021 Oct 24;10(21):4911. doi: 10.3390/jcm10214911 (PMC8584860; doi:10.3390/jcm10214911)
Supplement: Supplementary file 1 [file jcm-10-04911-s001.zip › jcm-1293689-supplementary.pdf]

Supplementary Table S1. Multivariate Cox regression analyses of futile events based on patients' baseline characteristics of preoperative factors. A hazard ratio (HR) above 1 increases the risk, below 1 decreases the risk of futility.

	**Combined access**				**TF-TAVR**				**TA-TAVR**			
	**HR**	**95% CI**		***p***	**HR**	**95% CI**		***p***	**HR**	**95% CI**		***p***
**Dichotomic parameters**												
Transapical access	1.569	0.987	2.493	0.057								
COPD	1.400	0.804	2.438	0.234								
Peripheral vascular disease	1.372	0.834	2.254	0.213					1.444	0.838	2.489	0.186
Cerebrovascular disease	1.797	1.095	2.950	**0.020**					2.066	1.187	3.598	**0.010**
Home oxygen dependence	2.500	0.766	8.160	0.129					5.591	1.620	19.295	**0.006**
Wheel chair dependence					4.976	1.188	20.844	**0.028**				
**Interval scaled parameters**												
Logistic EuroSCORE									1.000	0.973	1.028	0.980
EuroSCORE II	1.014	0.971	1.058	0.528					1.050	0.979	1.126	0.175
Postinterventional mean Gradient	1.008	0.980	1.037	0.587								
Postinterventional max. Gradient	0.983	0.962	1.005	0.125								

Supplementary Table S2. Multivariate Cox regression analyses of futile events based on patients' procedural characteristics. A hazard ratio (HR) above 1 increases the risk, below 1 decreases the risk of futility.

	**Combined access**				**TF-TAVR**				**TA-TAVR**			
	**HR**	**95% CI**		***p***	**HR**	**95% CI**		***p***	**HR**	**95% CI**		***p***
**Dichotomic parameters**												
Predillatation necessary									1.461	0.763	2.798	0.253
Conversion to open surgery	3.800	0.856	16.863	0.079					5.579	0.700	44.483	0.105
**Interval scaled parameters**												
Contrast medium, cc					1.001	0.994	1.008	0.743				
Procedure time, min									1.001	0.995	1.007	0.767
Total hours in the ICU	1.001	0.998	1.005	0.410	1.006	1.002	1.010	**<0.001**	1.001	0.997	1.005	0.696
Total hours ventilated	1.004	0.996	1.013	0.299					1.004	0.995	1.014	0.330
Length of stay after TAVR days	1.019	0.999	1.039	0.065					1.029	1.007	1.052	**0.011**

Supplementary Table S3. Multivariate Cox regression analyses of futile events based on patients' VARC-2 adverse events. A hazard ratio (HR) above 1 increases the risk, below 1 decreases the risk of futility.

	**Combined access**				**TF-TAVR**				**TA-TAVR**			
	**HR**	**95% CI**		***p***	**HR**	**95% CI**		***p***	**HR**	**95% CI**		***p***
**Dichotomic parameters**												
Device success	0.693	0.175	2.745	0.601					0.228	0.085	0.613	**0.003**
30-day combined safety endpoint	0.048	0.016	0.151	**<0.001**	0.120	0.046	0.311	**<0.001**	0.075	0.029	0.198	**<0.001**
Acute kidney injury	1.294	0.394	4.254	0.671					0.623	0.220	1.768	0.374
New atrial fibrillation	1.575	0.619	4.007	0.341					1.184	0.515	2.723	0.690
Reoperation for non-cardiac problems	1.925	0.514	7.214	0.331					0.776	0.301	2.003	0.601

Reoperation for bleeding/tamponade	0.706	0.171	2.921	0.631					1.668	0.497	5.592	0.407
Pneumonia under antibiotic treatment									2.648	0.836	8.394	0.098
Major bleeding complication	0.721	0.200	2.596	0.617	2.764	0.990	7.717	0.052	0.168	0.052	0.540	**0.003**
New renal replacement therapy	0.063	0.005	0.834	**0.036**					3.620	0.743	17.629	0.111
Major vascular complication									2.034	0.450	9.189	0.356
Neurological adverse event	0.179	0.018	1.782	0.142	0.486	0.059	3.995	0.502	0.736	0.134	4.057	0.725
Reoperation for valvular dysfunction									0.479	0.084	2.743	0.409
Myocardial infarction	1.042	0.105	10.355	0.972					31.137	4.329	223.932	**<0.001**
Mean gradient post-implant	0.800	0.629	1.016	0.068	0.910	0.755	1.098	0.325				
Max. gradient post-implant	1.082	0.932	1.257	0.301	0.967	0.849	1.102	0.618				
Max. flow post-implant	0.640	0.268	1.529	0.315	1.026	0.278	3.785	0.969				
